# Supplementary material for: Motor cortex signals for each arm are mixed across hemispheres and neurons yet partitioned within the population response
Source: eLife. 2019 Oct 9;8:e46159. doi: 10.7554/eLife.46159 (PMC6785221; doi:10.7554/eLife.46159)
Supplement: Transparent reporting form [file elife-46159-transrepform.docx]

***eLife’s* transparent reporting form**

We encourage authors to provide detailed information *within their submission* to facilitate the interpretation and replication of experiments. Authors can upload supporting documentation to indicate the use of appropriate reporting guidelines for health-related research (see [EQUATOR Network](http://www.equator-network.org/%20)), life science research (see the [BioSharing Information Resource](https://biosharing.org/)), or the [ARRIVE guidelines](http://www.plosbiology.org/article/info:doi/10.1371/journal.pbio.1000412) for reporting work involving animal research. Where applicable, authors should refer to any relevant reporting standards documents in this form.

If you have any questions, please consult our Journal Policies and/or contact us: [editorial@elifesciences.org](mailto:editorial@elifesciences.org).

**Sample-size estimation**

- You should state whether an appropriate sample size was computed when the study was being designed
- You should state the statistical method of sample size computation and any required assumptions
- If no explicit power analysis was used, you should describe how you decided what sample (replicate) size (number) to use

Please outline where this information can be found within the submission (e.g., sections or figure legends), or explain why this information doesn’t apply to your submission:

We recorded neural data from two nonhuman primates, which is the standard for the field. We did not perform a power analysis prior to recording. However, the number of units that we used (533 monkey E; 617 monkey F) is larger than the number recorded from prior studies of bi-hemispheric M1 activity (typically between 100-200 per monkey). Number of monkeys is discussed in the “Behavior” section of Materials and methods, and the number of units is discussed in the “Surgical procedures and neural recording” section.

**Replicates**

- You should report how often each experiment was performed
- You should include a definition of biological versus technical replication
- The data obtained should be provided and sufficient information should be provided to indicate the number of independent biological and/or technical replicates
- If you encountered any outliers, you should describe how these were handled
- Criteria for exclusion/inclusion of data should be clearly stated
- High-throughput sequence data should be uploaded before submission, with a private link for reviewers provided (these are available from both GEO and ArrayExpress)

Please outline where this information can be found within the submission (e.g., sections or figure legends), or explain why this information doesn’t apply to your submission:

Number of recording days is reported in the “Behavior” section of the Matrials and Methods. Cell inclusion criteria and number of recorded cells for each monkey is reported in the methods section “Surgical procedures and neural recording.” EMG recordings are discussed in the methods section “EMG recording.” For some analyses, more stringent selection criteria were used. These analyses and selection criteria are discussed in the following methods sections: “Firing-rate impact of small movements of the non-performing arm,” “Fano Factor,” and “LFADS”

**Statistical reporting**

- Statistical analysis methods should be described and justified
- Raw data should be presented in figures whenever informative to do so (typically when N per group is less than 10)
- For each experiment, you should identify the statistical tests used, exact values of N, definitions of center, methods of multiple test correction, and dispersion and precision measures (e.g., mean, median, SD, SEM, confidence intervals; and, for the major substantive results, a measure of effect size (e.g., Pearson's r, Cohen's d)
- Report exact p-values wherever possible alongside the summary statistics and 95% confidence intervals. These should be reported for all key questions and not only when the p-value is less than 0.05.

Please outline where this information can be found within the submission (e.g., sections or figure legends), or explain why this information doesn’t apply to your submission:

Statistical tests are discussed in the following sections of the Methods: “Individual neuron analyses”, “Firing-rate impact of small movements of the non-performing arm,” and “Population predictions,” “Comparing activity patterns across hemispheres,” and throughout the results. Significance values are always given exactly, the statistical test is specified, and sample size is included. In the case where p-values are less than 0.001, p-values are typically not reported exactly, as the exact level of significance at this level is less informative than considering relative signal magnitudes (as discussed in the text)

(For large datasets, or papers with a very large number of statistical tests, you may upload a single table file with tests, Ns, etc., with reference to sections in the manuscript.)

**Group allocation**

- Indicate how samples were allocated into experimental groups (in the case of clinical studies, please specify allocation to treatment method); if randomization was used, please also state if restricted randomization was applied
- Indicate if masking was used during group allocation, data collection and/or data analysis

Please outline where this information can be found within the submission (e.g., sections or figure legends), or explain why this information doesn’t apply to your submission:

Each monkey served as its own control.

For analyses of individual neurons, each neuron’s activity under one behavioral condition was compared to its own activity in a different behavioral condition, as discussed in the results sections: “Neurons are active during movements of either arm,” “Neural response patterns are limb-dependent,” and “Neuron-neuron correlations are limb-dependent” , and in the method sections: “Individual-neuron analyses” and “Firing-rate impact of small movements of the non-performing arm.” For some population analyses, neural activity during one condition was also compared to activity during a different condition, as discussed in the results sections: “Neural activity occupies different dimensions for movements of different arms,” “Linear decoders naturally separate signals related to the two arms,” and “Neural trajectories on single trials,” and the methods sections, “Dimensionality Reduction,” and “Predicting non-performing arm EMG.”

For some analyses of neural population activity in one hemisphere of each monkey was compared to activity in the other hemisphere of the same monkey, as discussed in the results sections “Population activity is isomorphic across hemispheres,” and “Neural trajectory tangling is low for both hemispheres,” and the methods sections “Comparison of muscle-activity predictions between hemispheres” and “Comparing activity patterns across hemispheres.”

**Additional data files (“source data”)**

- We encourage you to upload relevant additional data files, such as numerical data that are represented as a graph in a figure, or as a summary table
- Where provided, these should be in the most useful format, and they can be uploaded as “Source data” files linked to a main figure or table
- Include model definition files including the full list of parameters used
- Include code used for data analysis (e.g., R, MatLab)
- Avoid stating that data files are “available upon request”

Please indicate the figures or tables for which source data files have been provided:

Trial-averaged neural data, EMG data, single-trial LFADS data and analysis functions can be accessed at the following public online repository: <https://osf.io/859st/>
